# Stromal cell-mediated mitochondrial redox adaptation regulates drug resistance in childhood acute lymphoblastic leukemia

**DOI:** 10.18632/oncotarget.5528

**Published:** 2015-10-13

**Authors:** Jizhong Liu, Ashish Masurekar, Suzanne Johnson, Sohini Chakraborty, John Griffiths, Duncan Smith, Seema Alexander, Clare Dempsey, Catriona Parker, Stephanie Harrison, Yaoyong Li, Crispin Miller, Yujun Di, Zhumur Ghosh, Shekhar Krishnan, Vaskar Saha

**Affiliations:** ^1^ Children's Cancer Group, Institute of Cancer Science, Manchester Academic Health Sciences Centre, University of Manchester, Manchester, United Kingdom; ^2^ Bioinformatics Centre, Bose Institute, P-1/2 CIT Scheme, Kolkata, India; ^3^ Mass Spectrometry Service, Cancer Research UK Manchester Institute, University of Manchester, Manchester, United Kingdom; ^4^ Applied Computational Biology and Bioinformatics Group, Cancer Research UK Manchester Institute, University of Manchester, Manchester, United Kingdom; ^5^ Tata Translational Cancer Research Centre, Kolkata, India

**Keywords:** tumor microenvironment, oxidative stress, metabolic stress response, drug resistance, ALL

## Abstract

Despite the high cure rates in childhood acute lymphoblastic leukemia (ALL), relapsed ALL remains a significant clinical problem. Genetic heterogeneity does not adequately explain variations in response to therapy. The chemoprotective tumor microenvironment may additionally contribute to disease recurrence. This study identifies metabolic reprogramming of leukemic cells by bone marrow stromal cells (BMSC) as a putative mechanism of drug resistance. In a BMSC-extracellular matrix culture model, BMSC produced chemoprotective soluble factors and facilitated the emergence of a reversible multidrug resistant phenotype in ALL cells. BMSC environment induced a mitochondrial calcium influx leading to increased reactive oxygen species (ROS) levels in ALL cells. In response to this oxidative stress, drug resistant cells underwent a redox adaptation process, characterized by a decrease in ROS levels and mitochondrial membrane potential with an upregulation of antioxidant production and MCL-1 expression. Similar expanded subpopulations of low ROS expressing and drug resistant cells were identified in pre-treatment bone marrow samples from ALL patients with slower response to therapy. This suggests that the bone marrow microenvironment induces a redox adaptation in ALL subclones that protects against cytotoxic stress and potentially gives rise to minimal residual disease. Targeting metabolic remodeling by inhibiting antioxidant production and antiapoptosis was able to overcome drug resistance. Thus metabolic plasticity in leukemic cell response to environmental factors contributes to chemoresistance and disease recurrence. Adjunctive strategies targeting such processes have the potential to overcome therapeutic failure in ALL.

## INTRODUCTION

Modern chemotherapeutic regimens use ten or more different drugs over a 2–3 year period to treat childhood ALL. Though recurrences are now fewer, relapsed ALL remains the fifth most common malignancy in children [1]. The complex chemotherapy schedules and the underlying genetic heterogeneity of the disease have made it difficult to understand the biological mechanisms for the variations in response to therapy, and relapses occurs in all cytogenetic subtypes. Within all genetic subtypes of ALL, the most sensitive predictive factor of outcome is the level of minimal residual disease (MRD) in bone marrow aspirates at the end of the first month of therapy. Patients with high MRD levels have an increased risk of relapse [2]. This suggests that MRD represents drug resistant subclones selected by specific chemotherapy schedules; MRD cells survive and proliferate over time giving rise to recurrence. We recently reported the superiority of mitoxantrone over idarubicin, in the context of a randomised clinical trial in patients with relapsed ALL [3]. Both drugs were administered on the first two days of therapy but MRD levels after 4 weeks treatment were comparable in both arms, indicating that intrinsic drug resistance to idarubicin was not causal to outcome. However, patients treated with mitoxantrone experienced a higher degree of myelosupression for up to 12 months after administration, suggesting that therapeutic benefit was related to an increased toxicity to the bone marrow, indicating the role of environment mediated drug resistance (EMDR) in tumor treatment failure [4, 5].

The abnormal tumor microenvironment induces a collection of cellular stress responses and plays a major roles in determining the metabolic status and chemosensitivity in cancer cells [6]. BMSC protect cancer cells from chemotherapy by activating pro-survival signal pathways such as PI-3K/AKT [7–9]; or releasing chemoprotective factors such as asparagine [10], fatty acids [11] or cysteine [12]. Whether BMSC mediated chemoprotection facilitates the emergence of drug resistant subpopulations leading to the development of MRD; and if such cells are able to persist over the long duration of ALL therapy remains unknown. The diverse mechanisms identified for BMSC mediated drug resistance also suggest that targeting specific signalling molecules may not be sufficient to overcome BMSC mediated multidrug resistance, and common downstream survival mechanisms need to be identified.

Oncogenic signalling pathways converge to adaptive cancer cell metabolisms in order to support survival. Targeting these unique biochemical alterations in cancer cells as a potential therapeutic approach is yet to be fully exploited [6, 13]. Cancer cells have altered redox status [6, 13–15] and the modifications in the levels of ROS have been linked to radioresistance of cancer stem cells [15]. In this study we show that BMSC mediated multidrug resistance may occur through redox adaptation in ALL cells. By targeting antioxidant and anti-apoptotic capacity in leukemic cells, we identify a potential strategy to overcome therapeutic failure in ALL.

## RESULTS

### Soluble factors produced by BMSC induce a multidrug resistant phenotype in ALL cells

**Figure 1:**
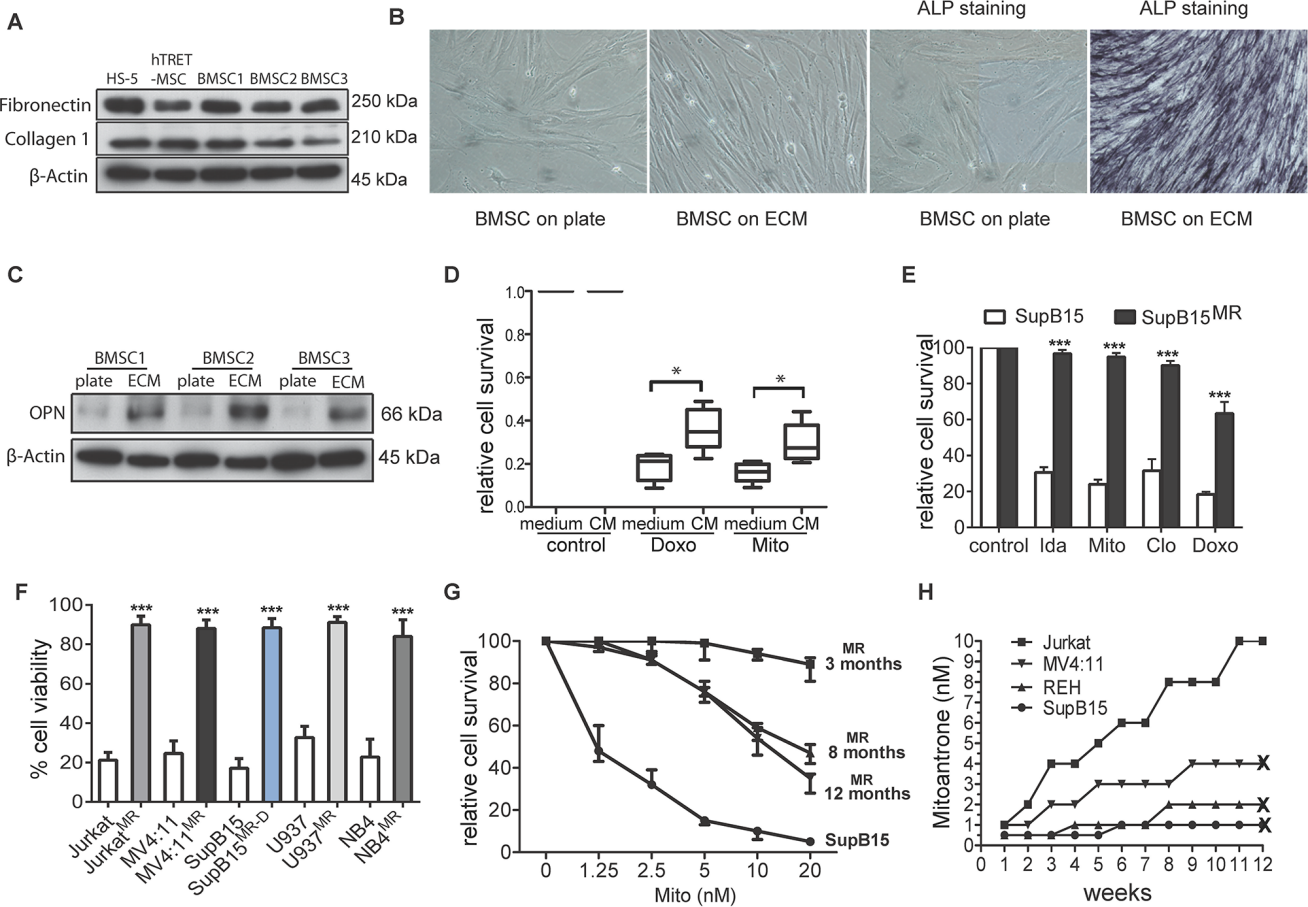
Generation of multidrug resistant subpopulations from ALL cell lines in a BMSC-ECM culture model **A.** BMSC-ECM scaffolds were generated from HS5, hTRET-BMSC, and primary BMSC from ALL patients (as BMSC1, 2, and 3); Immunoblots show the protein extracts from the ECM express fibronectin and collagen 1. β-Actin as loading control. **B.** Primary BMSC cultured on the BMSC-ECM scaffold showed filamentous spindle shaped morphology and alkaline phosphatase (ALP) positive staining (after 14 days culture). BMSC cells cultured in plate were set as control. Microscopy was performed with a Nikon TS100 Inverted Microscope at x20 magnification. **C.** When cultured on BMSC-ECM scaffold (ECM), primary BMSC have increased expression of osteopontin (OPN) compared to cells cultured on plate (BMSC-plate). β-Actin as loading control. **D.** BMSC derived CM protected primary ALL blasts from chemotherapy. Primary ALL blasts from 4 ALL patients were cultured in normal medium or CM, treated with doxorubicin (Doxo, 50 nM) or mitoxantrone (Mito 10 nM) for 3 days, cell survival were assessed by MTS assay. **E.** SupB15 and SupB15^MR^ cells were treated with idarubicin (Ida, 100 nM), Mito (10 nM), clofarabine (Clo, 300 nM), Doxo (50 nM) for 3 days. Cell survival was assessed by MTS assay. **F.** Jurkat^MR^, MV4:11^MR^, SupB15^MR-D^, U937^MR^, NB4^MR^ and their drug sensitive parent cells were treated with Mito (10 nM) for 3 days. Cell viability was assessed by trypan blue exclusion assay. **G.** Cell viability of SupB15 or SupB15^MR^ cells after treatment with increasing concentrations of Mito for 3 days. SupB15^MR^ cells which had been continuously cultured in drug-free medium for 8 or 12 months showed decreased drug resistant capacity. **H.** Jurkat, MV4:11, REH and SupB15 cells were incubated in normal medium and treated with stepwise dose increases in Mito (starting at 0.5–1 nM concentrations). Drug dose was increased when cells were observed to grow satisfactorily at a given dose level. At 3 months, only Jurkat cells survived the 10 nM of Mito; the other cell lines did not survive beyond 3 months at the indicated Mito doses. ‘X’, cell death. Data are mean ± SEM of at least three independent experiments (E,F). Non-parametric Mann-Whitney test (D) and unpaired 2-tailed Student's *t* test (E,F). *p < 0.05, ***p < 0.001.

Co-culture of ALL cells with BMSC promote survival, growth [16], and modulate the *in vitro* response to chemotherapy [17]. Such 2-D co-culture systems are being used to test efficacy of new drugs [18] and providing insights into the mechanisms of EMDR [19]. BMSC however exist in a complex 3-D milieu along with various types of extracellular matrix (ECM) [20, 21], and 3-D *in vitro* BMSC culture systems created on artificial or natural scaffolds have provided differential insights in the mechanisms of hematopoiesis and oncogenesis [22, 23]. We selected a BMSC-ECM culture model, by growing BMSC on a biological and physiologically relevant ECM scaffold [24] (Supplementary Figure S1A). Briefly, BMSC were cultured on the plate till confluent, treated with Triton X-100 and NH_4_OH, washed with PBS to remove cellular components, only ECM remained on the plate. The ECM scaffold was produced by BMSC, contained fibronectin and collagen I (Figure 1A), and facilitated BMSC differentiation into osteoblast-like cells (Figure 1B, 1C). The BMSC-ECM culture model contained key bone marrow components including ECM, BMSC, osteoblast-like cells, and factors released by BMSC and osteoblast-like cells.

*In vitro* BMSC mediated chemoprotection has been investigated by incubating cancer cells in BMSC derived conditioned medium (CM), or co-culturing cancer cells with BMSC, and then treating with drugs for 3 or 4 days [17, 19]. In the BMSC-ECM culture model, leukemia cells lines incubated in CM or long term co-cultured with BMSC (LTCC) showed a multi-drug resistant phenotype (Supplementary Figure S1B, S1C, S1D), a phenomenon also demonstrated by primary ALL cells (Figure 1D).

To mimic the effect of chemotherapy within the bone marrow microenvironment, ALL cell lines SupB15, REH, MV4:11 and Jurkat; acute myeloid leukemia cell line U937 and acute promyelocytic leukemia cell line NB4 cells were incubated in human BMSC cell line HS-5 derived CM, treated with 10 nM of mitoxantrone (Mito) for 6 days and then maintained in drug-free medium for 3 months. Control cells were incubated in normal medium and treated identically. This dose of drug was wholly lethal to cell in normal medium, but a population of leukemia cells incubated in CM survived the treatment and gave rise to multidrug resistant (MR) subpopulation. Similar MR cells were generated from SupB15 cells treated with doxorubicin (SupB15^MR-D^) (Figure 1E, 1F). BMSC releases small molecular weight chemoprotective molecules such as fatty acids [11] or cysteine [12]. Our results showed that both the <3kDa and ≥3kDa fraction of the CM are chemoprotective. On heating or after proteinase K treatment, CM continued to preserve its chemoprotective effects (Supplementary Figure S1E). However, neither the <3kDa nor the ≥3kDa fractions could generate MR clones from ALL cells lines (Supplementary Figure S1F), suggesting that the MR phenotype occurred as a result of multiple soluble factors present in CM.

SupB15^MR^ cells show partial restoration of chemosensitivity after 8 months of continuous culture in drug free medium (Figure 1G), indicated an epigenetic mechanism, previously described in drug resistant cell lines [25]. To further investigate the origin of the MR clones, SupB15, REH, MV4:11, or Jurkat cells were incubated in normal culture medium in the presence of 0.5 nM of MITO for 2 weeks and then with gradual increases in the Mito dose every 2–3 weeks. Cell viability was continuously monitored for 3 months. As shown in Figure 1H, drug resistant subclones were only generated from Jurkat cells. While SupB15, REH and MV4:11 cells survived treatment with 2 nM or 4 nM of Mito for a short time, they finally died out within 2–3 months. These results showed that BMSC protected ALL cells from chemotherapy in a cell type and drug-independent manner. This chemoprotection facilitated the emergence of reversible multidrug resistant subclones, which were more likely to be epigenetically derived [25].

### BMSC induces adaptation in ALL cells characterized by decreased pAKT and ROS levels and upregulation of MCL-1

**Figure 2:**
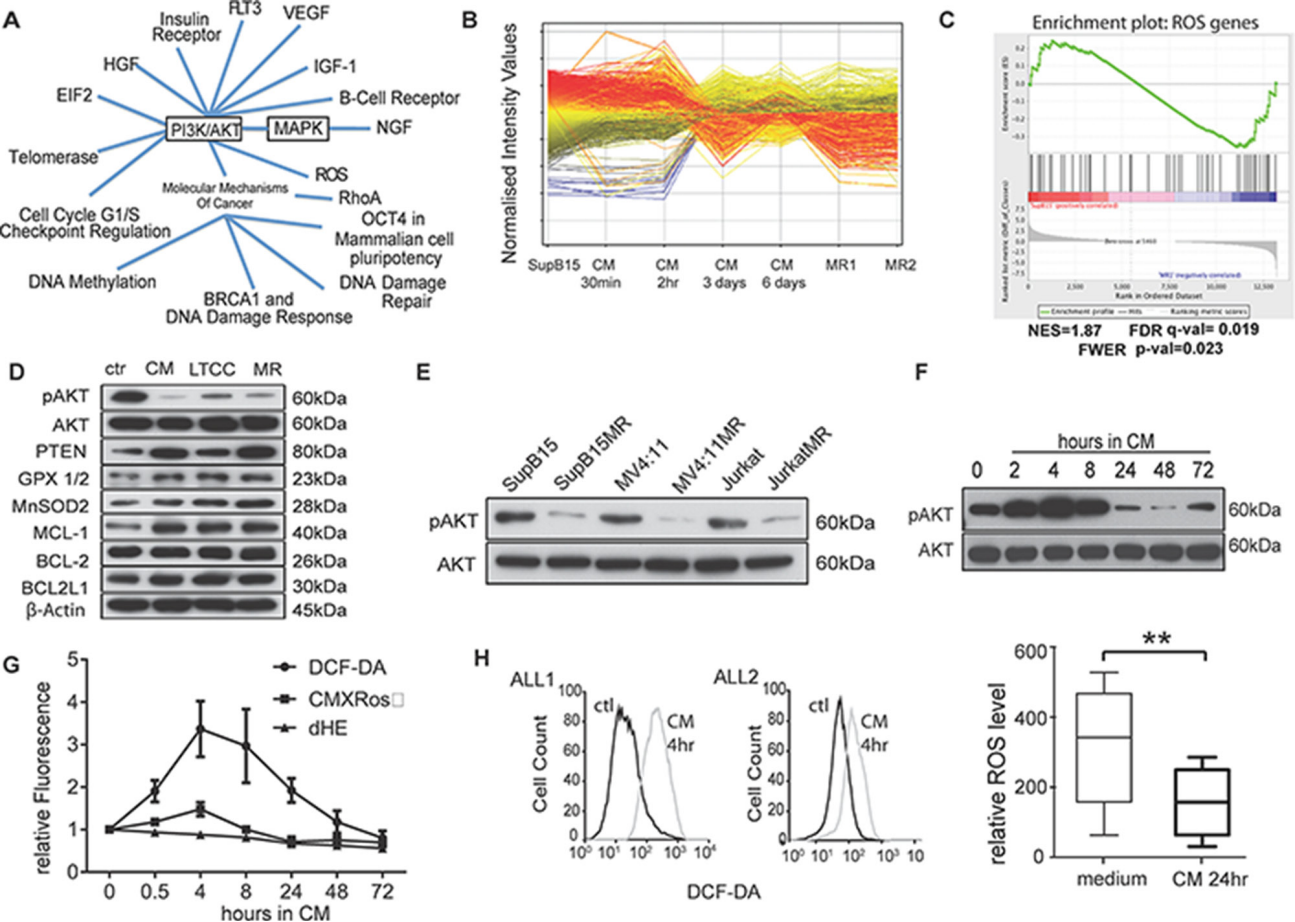
Multidrug resistant cells have lower intracellular ROS levels and AKT activity **A.** Ingenuity pathway analysis of phosphoproteomic data show signal pathways activated in SupB15 cells after exposure to HS-5 derived CM for 2 hours. **B.** Gene expression profiles of SupB15, SupB15 incubated in CM for different times, and two SupB15^MR^ clones (MR1 and MR2). Data were analysed by GeneSpring GX software (Agilent Technologies, Inc.). **C.** GSEA plot showing differential expression of ROS related genes in SupB15 and SupB15^MR^ cells. GSEA plots of gene expression related to AKT, apoptosis, chromatin remodeling, DNA repair, and ATPase are shown in Supplementary Figure S2D. **D.** Compared with SupB15 cells incubated in normal medium (ctr), SupB15 cells cultured in CM for 3 days, SupB15^LTCC^ and SupB15^MR^ show increased expression of PTEN, Glutathione peroxidase (GPX 1/2), MnSOD2 and MCL-1; decreased expression of ser473-AKT phosphorylation (pAKT), and relative unchanged expression of BCL-2 and BCL2L1. **E.** MR cells have decreased pAKT compared to their drug sensitive parent cells. All cells were maintained in normal medium. **F.** Immunoblot showing temporal biphasic expression of pAKT in SupB15 cells incubated in CM. Following an initial increase (2–8 hours incubation), pAKT expression declines (24–48 hours incubation), then stabilizes to a lowered steady state at 72 hours. **G.** ROS level (DCF-DA), Superoxide level (Dihydroethidium, dHE), and Δψm (CMXRos) in SupB15 cells when incubated in CM for 0.5 to 72 hours, cells incubated in normal medium as time ‘0′. **H.** ROS levels increased in primary ALL blasts after incubated in medium containing 50% of CM (v/v) for 4 hours (*n* = 2, histogram); and decreased after 24 hours' incubation (*n* = 6, box plot). Cells incubated in normal medium as control (ctl). Data are mean ± SEM of at least three independent experiments. Non-parametric Mann-Whitney test (H) **p < 0.01.

In this study, BMSC mediated short-term (CM protected leukemia cells from 3–4 days' treatment) and long-term (CM help to generate drug resistant subclones) drug resistance. To investigate the mechanism underlying BMSC induced drug resistance, we first used SILAC-based phosphoproteomics to investigating the changing of signalling pathway activity in short-term (30 minutes and 2 hours) CM-exposed SupB15 cells (Supplementary Figure S2A). 1295 differentially phosphorylated peptides, attributable to 720 known proteins were identified after 2 hours' exposure to CM (Supplementary Table S1). Ingenuity pathway analysis (www.qiagen.com/ingenuity) identified activation of PI3K/AKT, MAPK and ROS pathways in SupB15 cells among others (Figure 2A and Supplementary Figure S2B). Chemosensitivity could be restored on prior exposure to PI3K inhibitors (Supplementary Figure S2C), suggesting that an alteration in PI3K signalling was contributory to the short term drug resistant phenotype.

Comparative gene expression analysis were performed on normal cultured SupB15; SupB15^MR^; and SupB15 cells incubated in CM for different times (from 30 minutes to 6 days) (Figure 2B). Functional annotation and gene set enrichment analysis of the 6,357 genes differentially expressed between normal cultured SupB15 and the other treatment groups, revealed significant association with regulation of ROS, PI3K-AKT, MAPK, apoptosis and cell cycle checkpoint pathways (Figure 2C and Supplementary Figure S2D, S2E; Supplementary Tables S2 and S3). Based on this functional information, a heatmap with the representative gene set (2489 genes) confirmed the distinct contrast in gene expression signature between MR clones and their corresponding wild type control (Supplementary Figure S2F). Principal component analysis identified two distinct clusters with similarity between SupB15^MR^ and SupB15 cells exposed to CM for 3 or 6 days (Supplementary Figure S2G). This suggested a temporal switch in drug resistant pathways between 3–6 days of exposure to CM. Verification of the identified pathways using Western blot showed that drug resistant cells, including MR cells, SupB15 cells incubated in CM for 3 days, and SupB15^LTCC^ cells, had increased expression of antioxidants (MnSOD2, GPX1/2), anti-apoptotoic protein MCL-1, and decreased AKT ser473 phosphorylation when compared to cells grown in normal medium (Figure 2D). Low pAKT was also detected in Jurkat^MR^ and MV4:11^MR^ cells (Figure 2E).

Time course experiments were performed to investigate the effects of CM on AKT activity in ALL cells. As shown in Figure 2F, AKT activity in SupB15 cells fluctuated according to incubation time. After exposure to CM, AKT ser-473 phosphorylation increased at 2–8 hours with a subsequent decline from 24 hours to a steady level at 72 hours. Intracellular ROS level in ALL cells showed similar fluctuations after exposure to CM. In SupB15 cells, ROS levels and mitochondrial membrane potential (Δψm) increased from 0.5 hours, peaked at 4 hours, and decreased to lower than basal levels at 72 hours (Figure 2G). In primary ALL blasts, ROS levels increased after 2 hours' exposure to CM, but decreased after 24 hours (Figure 2H). MR cells too have lower intracellular ROS levels and Δψm when compared with their drug sensitive parent cells (Supplementary Figure S2H, S2I).

### BMSC induced chemoresistance is distinct from its effect on cellular proliferation

**Figure 3:**
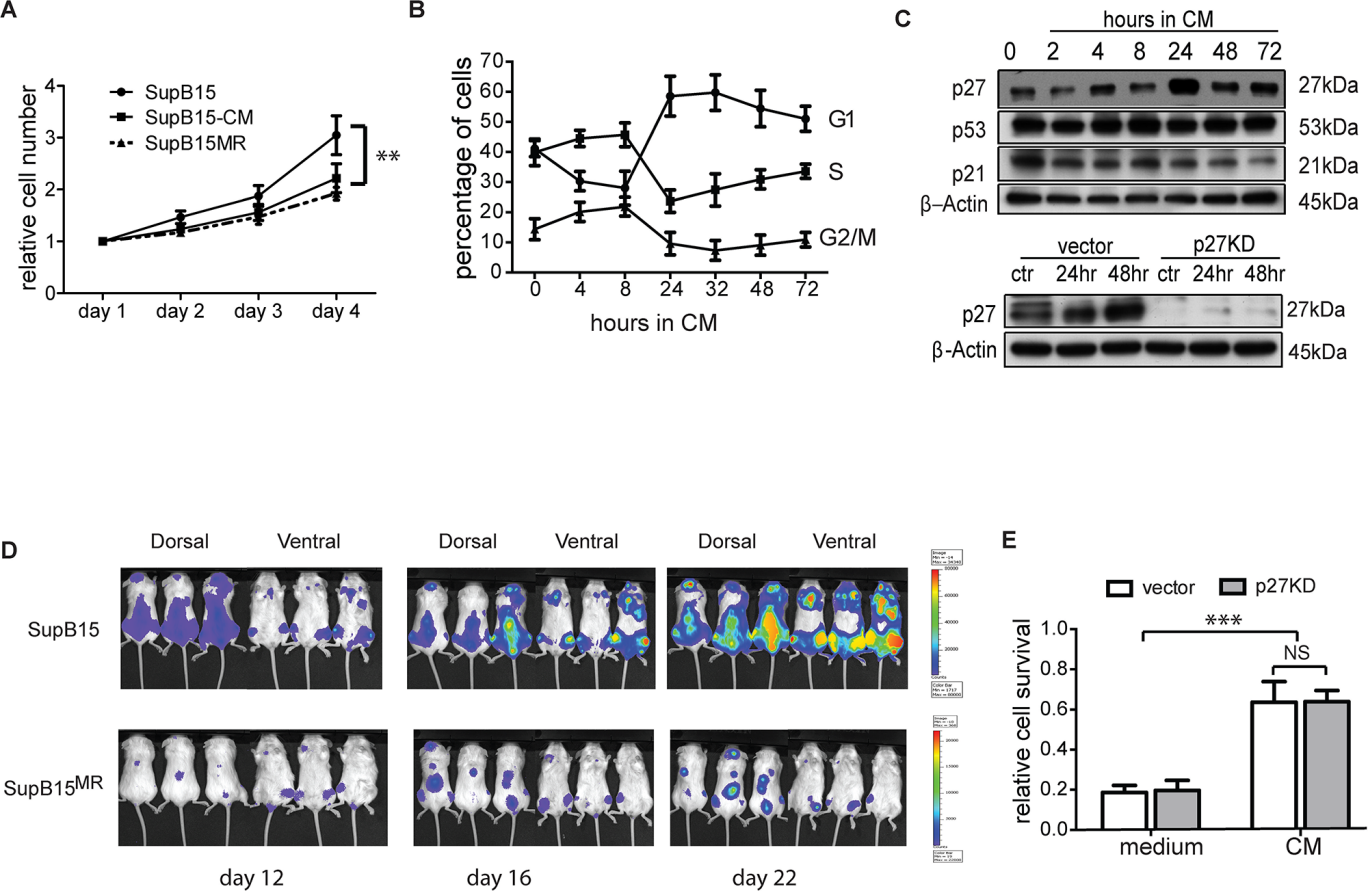
BMSC induced chemoresistance is distinct from its effect on cellular proliferation **A.** SupB15 and SupB15^MR^ cells were cultured in normal medium; or SupB15 cells were maintained in medium containing 50% of CM (v/v) for 4 days. Cell number were normalised to day 1. Cell proliferation was assessed with MTS assay. **B.** Cell cycle progression in SupB15 cells when cultured in CM. After 24 to 72 hours, cells showed a higher proportion of cells in G1 phase, lower proportion of cells in S and G2/M phase. **C.** After incubation in CM for 24 to 72 hours, SupB15 cells showed increased expression of p27, decreased expression of p21, the expression of p53 did not change (upper panel). CM failed to upregulate p27 expression in SupB15^p27KD^ cells (lower panel) after 24 or 48 hours' incubation. **D.** 1 × 10^5^ of SupB15^LucGFP^ or SupB15^MRLucGFP^ cells were injected into NSG mice (*n* = 6 each group) intravenously. Representative images of 3 mice from a cohort of 6 animals showing delayed engraftment kinetics of SupB15^MR^ transplanted mice when comparing with SupB15 cells. Leukemia engraftment was measured with bioluminescence. **E.** SupB15 cells with p27 wild type (vector) or knockdown (p27KD) were incubated in normal medium or CM. CM equally protected SupB15^vector^ and SupB15^p27kd^ cells from Doxo (50 nM, 72 hours) treatment. Data are mean ± SEM of at least three independent experiments (A,B,E). unpaired 2-tailed Student's *t* test (A, E). **p < 0.01, ***p < 0.001.

Constitutive activation of the PI3K/AKT pathway promotes cancer cell proliferation [26–29]. The low AKT activity mediated by BMSC in ALL cells was associated with decreased cellular proliferation (Figure 3A) and cell cycle progression (Figure 3B), and increased p27 expression (Figure 3C) *in vitro*. Comparing with SupB15 cells, SupB15^MR^ showed delayed engraftment kinetics in NOD-*scid* IL2Rgamma^null^ (NSG) mice (Figure 3D). This is consistent with evidence that BMSC inhibit proliferation and function of immune cells [30, 31], as ALL cells are malignant counterparts of immature lymphocytes.

To assess the association between BMSC mediated growth suppression and chemoprotection, SupB15 cells were stably transduced with p27shRNA (SupB15^p27kd^) or vector control (SupB15^vector^), incubated in CM or normal medium and treated with doxorubicin for 3 days. Unlike that observed from SupB15^vector^ cells, SupB15^p27kd^ cells grew at a similar rate when cultured in CM or normal medium (Figure 3C, lower panel; and 3E). Nevertheless, CM was equally protective of SupB15^p27kd^ or SupB15^vector^ cells against doxorubicin (Supplementary Figure S3A). When incubated in CM, SupB15 cells have high AKT activity at 2 hours and low AKT phosphorylation after 24 hours (Figure 2F). The proportion of cells at G1 phase was increased significantly after 24 hours incubation (Figure 3B). 10 nM of Mito was added into culture at these two time points (2 hours and 24 hours after incubation in CM). After 3 days' treatment, cell survivals in these two groups were similar (Supplementary Figure S3B). These results suggested that BMSC-ECM mediated chemoprotection is not regulated by alterations in cellular proliferation.

### BMSC regulates intracellular ROS level and induce redox adaptation in ALL cells

**Figure 4:**
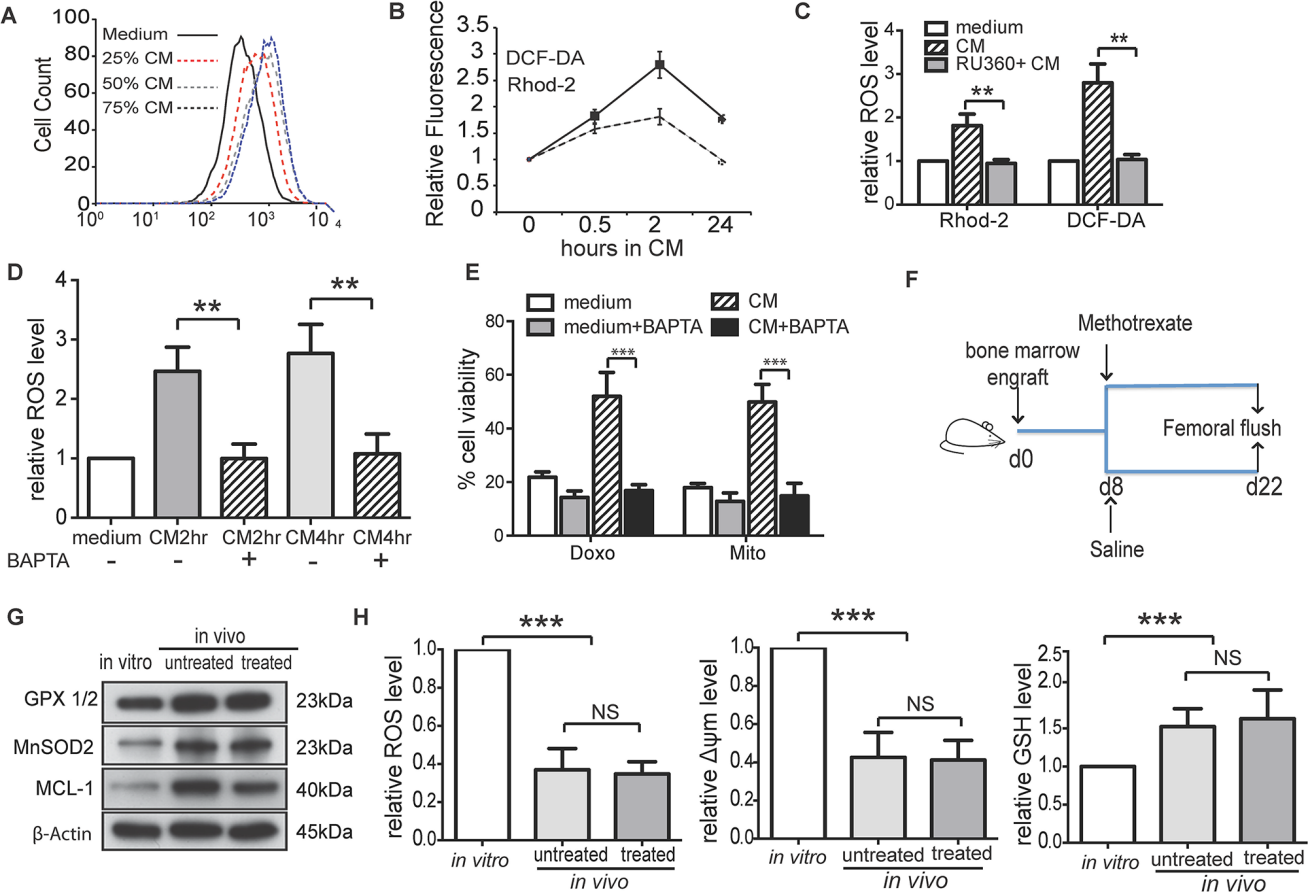
BMSC regulates intracellular ROS levels and induce redox adaptation in ALL cells **A.** Medium containing different proportion of CM (25%, 50% or 75%, v/v) increased ROS production in REH cells after 4 hours' incubation. **B.** Level of ROS (DCF-DA) and mitochondrial calcium (Rhod-2) in SupB15 cells after incubation in CM for indicated times. **C.** SupB15 cells were treated with RU360 (10 μM) for 1 hour, then incubated in CM for 2 hours. CM failed to increase ROS level. **D.** and **E.** Pre-treatment of SupB15 cells with BAPTA-AM (3 μM, 2 hours) inhibited (D) CM induced ROS generation and (E) CM mediated chemoprotection. **F.** and **G.** Intrafemorally engrafted SupB15^LucGFP^ cells showed increased expression of GPX1/2, MnSOD2, and MCL-1 when compared to SupB15^LucGFP^ cells cultured *in vitro*. **H.** Intrafemorally engrafted SupB15^LucGFP^ cells had lower levels of ROS and Δψm; higher level of GSH when compared to *in vitro* cultured cells. *n* = 6 mice per group. Data are mean ± SEM of at least three independent experiments (C,D,E). Non-parametric Mann-Whitney test (H) and unpaired 2-tailed Student's *t* test (C,D,E). **p < 0.01, ***p < 0.001.

Compared with cells in normal medium, medium containing 25% CM (v/v) also increased ROS levels (Figure 4A), suggesting that the increased ROS production in ALL cells was not due to the lack of nutrients in CM. ROS generating enzymes are activated by increased mitochondrial calcium (mCa^2+^) concentrations [32]. To examine whether BMSC regulation of ROS metabolism was related to mCa^2+^ influx, SupB15 cells were incubated in CM and mCa^2+^ concentrations were assessed by Rhod-2 staining followed with flow cytometry analysis. mCa^2+^ concentrations increased after 30 minutes, and gradually decreased from 2 hours returning to basal levels after 24 hours. Intracellular ROS levels paralleled the temporal changes in mCa^2+^ concentrations (Figure 4B). The fluctuations of mitochondrial calcium influx were unrelated to intercellular Ca^2+^ concentrations as they were similar in both CM (30.6 ± 2.42mg/L) and normal medium (30.8 ± 0.8mg/L). RU360, a selective inhibitor of mitochondrial calcium uptake, suppressed CM induced ROS generation (Figure 4C). As RU360 is unstable in the presence of oxygen, the calcium chelator BAPTA-AM was used to test the role of mCa^2+^ influx in CM induced drug resistance. CM failed to stimulate ROS generation (Figure 4D) or provide chemoprotection in BAPTA-AM pre-treated ALL cells (Figure 4E).

We have shown that BMSC-CM dynamically regulated ROS levels in ALL cells and resulted in a low intracellular ROS status. The decreased ROS levels in ALL cells after incubation in CM was not the consequence of CM mediated growth inhibition, as SupB15^p27kd^ cells have the same proliferation rate when cultured in normal or CM, but ROS levels decreased after incubation in CM for 72 h (Supplementary Figure S4). These findings suggested that the BMSC microenvironment induced a redox adaptation in ALL cells. To address this hypothesis, NSG mice were engrafted intrafemorally with 1 × 10^5^ of SupB15^LucGFP^ cells and treated with methotrexate (5 mg/kg) or saline from day 8 (Figure 4F). Compared with *in vitro* cultured cells, SupB15^LucGFP^ cells obtained from femoral flushes of both treated and untreated mice showed higher expression of GPX1/2, MnSOD2 and MCL-1 (Figure 4G), lower levels of ROS and Δψm, and higher levels of GSH (Figure 4H). Together, BMSC microenvironment induces an initial increase in intracellular ROS levels and Δψm via an influx of calcium ions into mitochondria. As excess ROS is harmful to cells, ALL cells adapt to this oxidative stress by increasing antioxidant production and lowering intracellular ROS levels. This redox-adapted phenotype is maintained by cells surviving post exposure to chemotherapy, as illustrated by MR cells.

### Low ROS levels characterize subpopulations of primary ALL cells with relative resistance to chemotherapy

**Figure 5:**
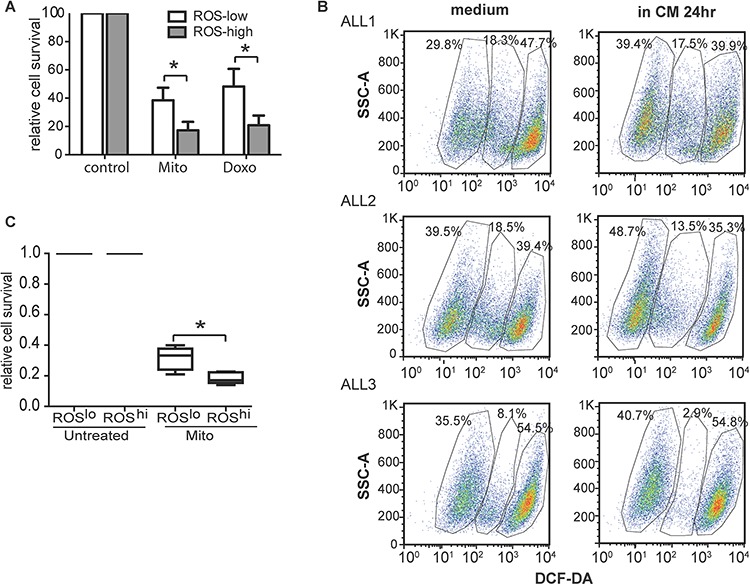
Low ROS levels characterized subpopulations of primary ALL cells with relative resistance to chemotherapy **A.** Ros^low^ SupB15 cells were relatively drug resistant than that of Ros^high^ cells after treated with Mito (10 nM) or Doxo (50 nM) for 72 hours. **B.** Flow cytometry dot plots (side scatter SSC *vs* DCF-DA staining) indicated presence of distinct ROS^high^, ROS^intermediate^ and ROS^low^ subpopulations in CD19 positive primary ALL cells (ALL1, ALL2, ALL3). The ROS^low^ subpopulation expanded on 24-hours incubation with CM. Lymphoprep™ density gradient centrifugation were used to exclude dead cells. After 24 hours' incubation in normal medium or CM, cell viability were more than 90% (Trypan blue exclusive assay), and cells incubated in CM had better viability than cells incubated in normal medium. Dead cells were further excluded by SSC/FSC gating on Flowjo software. **C.** ROS^low^ populations of primagraft ALL blasts were more drug resistant than that of ROS^high^ cells (*n* = 5). CD19 positive primagraft cells were collected from mice bone marrow flushes, purified with magnetic purification, and dead cells were excluded by Lymphoprep™ density gradient centrifugation. Cells were sorted into ROS^low^ and ROS^high^ cells based on intracellular ROS level (DCF-DA staining). In the process of cell sorting, dead/dying cells were excluded by SSC/FSC gating and propidium iodid (PI) staining. Cells were then incubated in medium containing 50% CM (CM), and treated with Mito (15 nM) for 72 hours. Data are mean ± SEM of at least three independent experiments. unpaired 2-tailed Student's *t* test (A) and Non-parametric Mann-Whitney test (C) *p < 0.05, ***p < 0.001.

Both long term CM-exposed ALL cells and MR cells had low ROS levels. We proposed that ROS^low^ subpopulations of CM-exposed ALL cells were likely to be more resistant to chemotherapy than ROS^high^ subpopulations. To test this, SupB15 cells were incubated in CM for 6 days and sorted into ROS^low^ and ROS^high^ populations (<10% of whole population) using DCF-DA staining. ROS^low^ cells were relative resistant to chemotherapy with Mito or Doxo when compared to ROS^high^ cells on (Figure 5A). Next we examined the chemosensitivity of the subpopulations in primary ALL blasts with differential intracellular ROS levels. CD19+ primary ALL blast cells, obtained from diagnostic bone marrow aspirates could be characterized into 3 subpopulations based on ROS levels, low, medium and high. After exposure to CM for 24 hours, there was a moderate expansion in the ROS^low^ subpopulation (Figure 5B). We engrafted primary ALL cells intrafemorally in non-irradiated NSG mice. Femoral flushes were obtained on evidence of engraftment and purified CD19+ ALL blasts were sorted into ROS^low^ and ROS^high^ populations, treated with Mito or Doxo for 3 days. ROS^low^ cells were again shown to be comparatively more drug resistant than ROS^high^ cells (Figure 5C).

### Inhibiting antioxidants and antiapoptotic capacity overcomes BMSC mediated drug resistance

**Figure 6:**
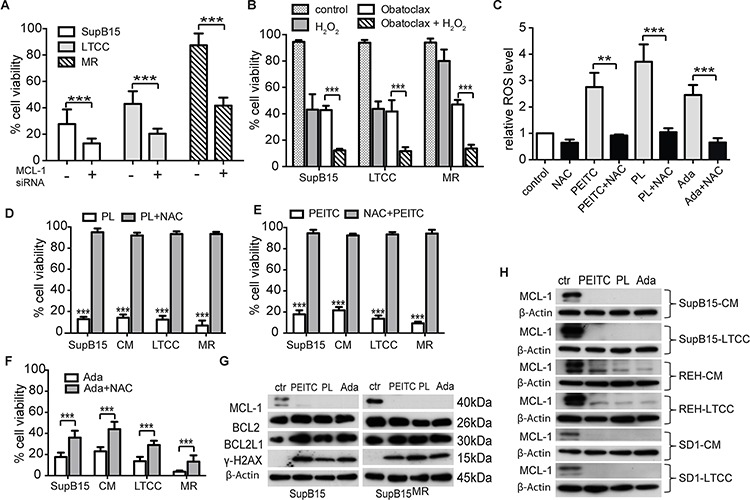
Inhibiting antioxidants and antiapoptotic capacity overcome BMSC mediated drug resistance **A.** and **B.** Suppression of MCL-1 expression sensitizes SupB15 cells to H_2_O_2_ treatment. SupB15 cells were (A) transfected with MCL-1 siRNA (1 μM) for 72 hours; or (B) treated with Obatoclax (100 nM) for 24 hours. Cells were then treated with H_2_O_2_ (80 μM) for 24 hours. Cell viability was assessed by trypan blue exclusion assay. **C.** PEITC (5 μM), PL (10 μM), or Ada (5 μM) stimulated ROS production in SupB15^MR^ cells after 1 hour treatment. Effects were reversed by NAC (3 mM, 2 hours) pretreatment. **D, E.** and **F.** PEITC (D), PL (E) and Ada (F) were toxic for SupB15 in normal medium (as control); SupB15 incubated in CM; SupB15^LTCC^; and SupB15^MR^ cells. Note the differential effects of NAC (3 mM) pre-treatment on the toxicity of PEITC, PL and Ada. **G.** 2 hours of PEITC (5 μM), PL (10 μM), or Ada (5 μM) treatments inhibited MCL-1 expression, and induced γ-H2AX expression in SupB15 and SupB15^MR^ cells. Cells were maintained in normal medium. **H.** MCL-1 expressions in cells were strongly inhibited after 2 hours of PEITC (5 μM), PL (10 μM), or Ada (5 μM) treatment: SupB15, REH and SD1 cells were incubated in CM for 3 days; and SupB15^LTCC^, REH^LTCC^, and SD1^LTCC^ were cultured in normal medium. Data are mean ± SEM of at least three independent experiments. unpaired 2-tailed Student's *t* test (A,B,C,D,E,F). **p < 0.01, ***p < 0.001.

As shown earlier, the drug resistant phenotype was further characterized by upregulation of the antiapoptotic BCL2 family members BCL-2, BCL2L1 and MCL-1. Overexpression of MCL-1 has been previously associated with drug resistance in cancer [33–35]. MCL-1 was upregulated in LTCC, CM exposed ALL cells, MR cells (Figure 2D), and bone marrow engrafted primary ALL cells (Figure 4G). Transient MCL-1 knockdown by siRNA, or the MCL-1 inhibitor Obatoclax sensitized SupB15 and SupB15^MR^ cells to H_2_O_2_ cytotoxicity (Figures 6A, 6B, and Supplementary Figure S5), suggesting that MCL-1 contributes to the redox adaptation process in ALL.

Under oxidative stress, cancer cells increase their antioxidant capacity and develop a drug resistant phenotype. Targeting this redox adaptation process has significant therapeutic implications [13]. Piperlongumine (PL) [36, 37], β-phenethyl isothiocyanate (PEITC) [38] and Adaphostin (Ada) [39] have dual effects of increasing ROS production and suppressing BCL-2 family molecules. 10 μM of PEITC, PL, or Ada strongly stimulated ROS generation (Figure 6C) and were lethal to SupB15^MR^, SupB15^LTCC^ and SupB15^CM^ cells (Figure 6D, 6E and 6F). The ROS scavenger N-acetyl-cysteine (NAC) reversed the effects of PEITC or PL, but was less effective against Ada. This may due to the fact that Ada inhibits ABL1 kinase activity and SupB15, while highly chemosensitive, is a BCR-ABL positive cell line. All three drugs strongly induced γ-H2AX phosphorylation and inhibited MCL-1 (not BCL-2 or BCL2L1) expression (Figure 6G and 6H). Therefore the simultaneous increase of ROS and inhibition of MCL-1 appeared to inhibit BMSC induced chemoprotection.

### Targeting antioxidants and MCL-1 effectively against ASNase resistance in primary ALL blasts

**Figure 7:**
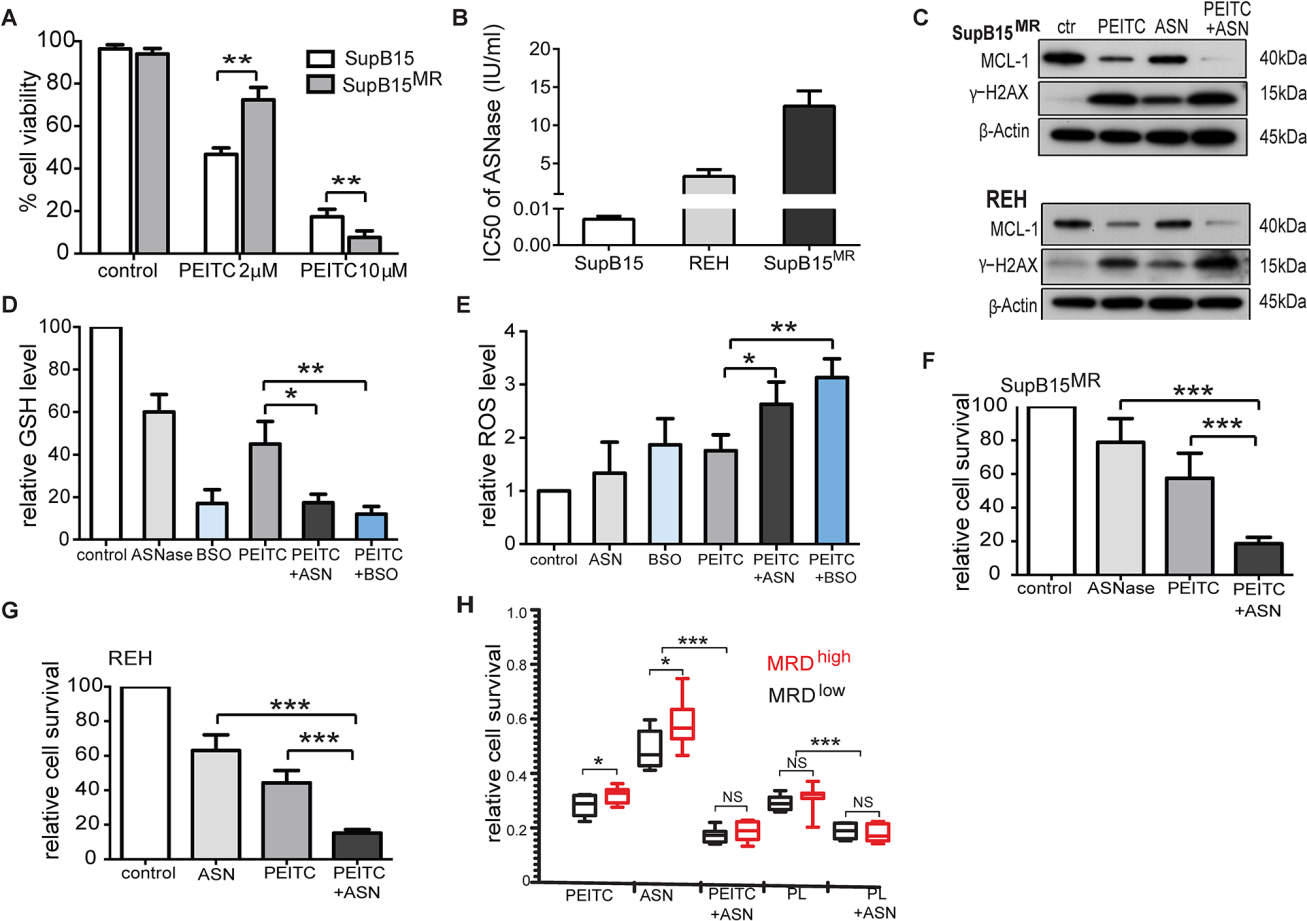
Targeting antioxidants and MCL-1 effectively restored sensitivity to ASNase in ALL cells **A.** Variations in cell viability of SupB15 and SupB15^MR^ cells after treatment with PEITC (2 μM or 10 μM) for 24 hours. **B.** IC50 of ANSase in SupB15, REH, and SupB15^MR^ cells. **C.** Combination of PEITC with ASNase strongly inhibited MCL-1 expression and increased γ-H2AX expression in SupB15^MR^ and REH cells. PEITC (2 μM) for 48 hours; ASNase (ASN) for 72 hours (2 IU/ml for REH, 5 IU/ml for SupB15^MR^); or ASNase for 24 hours, then PEITC for further 48 hours (PEITC + ASN). **D.** and **E.** Combination of PEITC with ASNase (ASN) or BSO (D) inhibited GSH expression and (E) increased ROS generation in SupB15^MR^ cells after 20 hours' treatment. PEITC (2 μM); ASNase (5 IU/ml); BSO (100 uM). **F.** and **G.** Cell viability of SupB15^MR^ (F) or REH (G) following treatment: PEITC (2 μM) 48 hours; ASNase 96 hours (5 IU/ml for SupB15^MR^, 2IU/ml for REH); ASNase 48 hours, then PEITC 48 hours (PEITC + ASN). **H.** The addition of PEITC or PL overcame ASNase resistance in primary ALL cells. Diagnostic blast cells from MRD^high^ or MRD^low^ patients were incubated in medium containing 50% CM, and treated with ASNase (2 IU/ml) for 72 hours; PEITC or PL (2 uM) for 48 hours; or ASNase for 24 hours, followed by PEITC or PL for further 48 hours. Data are mean ± SEM. Unpaired 2-tailed Student's *t* test (A,D,E,F and G) and nonparametric Mann-Whitney test (H) *p < 0.05, **p < 0.01, ***p < 0.001.

We next evaluated the role of antioxidant and MCL-1 targeting as adjunct strategies in ALL therapy. The anticancer effects of PEITC are dose-dependent. Compared to SupB15 cells, SupB15^MR^ were sensitive to 10 μM of PEITC but more resistant to 2 μM of PEITC (Figure 7A). Serum concentrations of PEITC reached ~1 μM after oral intake [40], posing a practical hurdle for its clinical use. GSH is the main cellular antioxidant. A potential strategy would be to combine PEITC with reagents that inhibit GSH synthesis, such as buthionine sulfoximine (BSO) or the anti-leukemic drug L-Asparaginase (ASNase). ASNase converts asparagine to aspartate and glutamine to glutamate. The depletion of both asparagine and glutamine by ASNase appears to be required for its cytotoxic action [41], and ASNase has been reported to deplete serum glutamine and suppresses GSH levels in ALL patients [42]. We speculated that the effect of ASNase could in part be related to its antioxidant activity and used in combination with PEITC could potentiate its effect. REH and SupB15^MR^ cells are highly resistant to ASNase when compared to SupB15 (Figure 7B). The combinations of PEITC with BSO or ASNase were more effective in suppressing MCL-1 activity, stimulating γ-H2AX expression (Figure 7C), inhibiting GSH expression (Figure 7D) and increasing ROS levels (Figure 7E) in SupB15^MR^ and REH cells, than PEITC or ASNase alone. PEITC potentiates ASNase cytotoxicity in SupB15^MR^ and REH cells (Figure 7F and 7G).

As shown earlier, in patients with ALL, BMSC microenvironment mediated redox adaptation has occurred in a proportion of leukemic cells prior to treatment. We speculated that these cells persist after initial therapy. Sufficiently expanded, these cells may give rise to detectable MRD. Primary diagnostic blasts obtained from MRD^low^ (<10^−4^) and MRD^high^ (≥10^−4^) patients recruited to the UKALL2003 clinical study [43] were analyzed (Supplementary Figure S6A). These patients were treated with a 3-drug induction of dexamethasone, vincristine and ASNase. 28 days after starting therapy, MRD assessments were performed. Trough ASNase activity was measured at two time points during induction (Supplementary Figure S6B). All patients had adequate ASNase activity at both time points (>100 U/L) [44], indicating adequate drug activity. As shown in Figure 7H, blast cells from MRD^hi^ (*n* = 10) patients displayed proportionately increased resistance to ASNase when compared to cells from MRD^low^ patients (*n* = 15). This is indicative of an expanded drug resistant population in the pre-treatment bone marrow sample in the MRD^hi^ group. The addition of PEITC or PL overcame ASNase resistance. Immunoblots showed that the combination of PEITC or PL with ASNase strongly inhibited MCL-1 levels and induced γ-H2AX expression in primary ALL blasts (Supplementary Figure S6C). Thus targeting antioxidants production and inhibiting MCL-1 may be potential adjunctive therapeutic strategies to overcome MRD in childhood ALL.

## DISCUSSION

**Figure 8:**
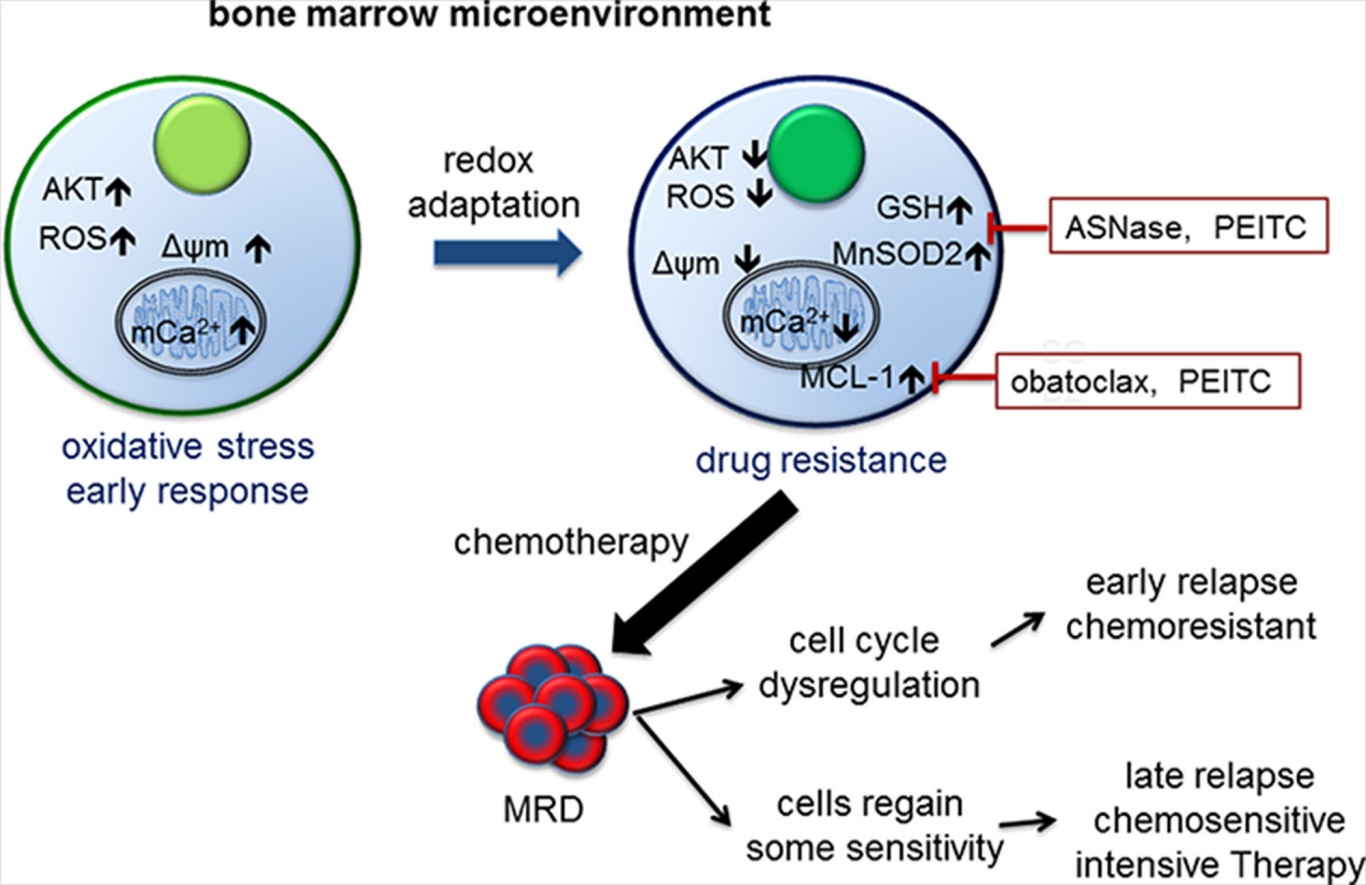
Model of bone marrow microenvironment mediated drug resistance in ALL and its clinical correlation The bone marrow microenvironment exerts oxidative stress on leukemic cells by promoting mitochondrial calcium influx, increasing intracellular ROS levels and Δψm, and activating AKT. In response, leukemic cells initiate a mediated redox adaptation process, resulting in an increased expression of antioxidants (e.g. GSH and MnSOD2) and MCL-1, decreased ROS levels, Δψm and AKT activity. This confers a multiple drug resistance (MR) phenotype in leukemic cells. MR cells exist at diagnosis and can be detected as MRD post therapy. Cells with deregulated cell cycle control give rise to early recurrence. Other drug resistant cells gradually regain a degree of chemosensitivity, and respond to intensification with cytotoxic drugs. Alternatively, inhibiting antioxidants and MCL-1 activity as adjunctive therapy can target the MR phenotype.

Analyses of the effect of the microenvironment created by the *in vitro* BMSC-ECM 3-D model offer possible biological explanations for the clinical variations in response to therapy in childhood ALL (Figure 8). As in most cancers, at diagnosis the majority of leukemic cells though metabolically active are not proliferative and mainly dying. Only a small populations of slowly cycling cells survive and give rise subsequently to fast cycling progenitors [45]. Our study suggests that the BMSC microenvironment produces an oxidative stress response in ALL cells. A subpopulation of ALL cells underwent redox adaptation, tolerated lethal doses of chemotherapy and gave rise to MR cells; a phenomenon not recapitulated by cells grown in normal medium. MR cells are characterised by decreased proliferation and multidrug resistance. This result agrees with the finding that in childhood ALL patients after 8 days treatment, persisting blast cells had decreased proliferative activity [46]. The method of generating MR cells parallels the clinical setting as cancer cells were cultured in a protective environment and exposed to toxic drug levels. In patient, treated with intensive combination chemotherapy MRD of ≥10^−4^ after 4 weeks of treatment is associated with a poorer outcome even though treatment lasts over 2-years. Further intensification with myelosuppressive therapy is associated with a clearance of MRD and better outcomes [47]. Our model suggests that EMDR derived, slowly proliferative, multidrug resistant cells survived initial cytotoxic stress and if sufficiently expanded were detectable as MRD. Cytotoxic therapy is non-specific and injurious to both leukemic and the protective microenvironment. This is one possible explanation for our observation of the clinical benefits of the more myelotoxic mitoxantrone when compared to idarubicin [3].

When removed from the stressful environment, MR cells gradually lose their drug resistant phenotype, a feature also described in other cancers [25] and provides a model for late relapses. ALL patients with late relapses occurring many months after stopping therapy often respond to further intensification by similar chemotherapeutic agents. If MRD is cleared then many patients can be cured second time round [47]. On the contrary recurrences whilst on therapy are often associated with rapidly progressive disease and poor outcomes even after allogeneic stem cell transplantation [3]. SupB15 cells with p27 knockdown retained their drug resistant phenotype but were proliferative when incubated in BMSC CM. Similarly, mutations and deletion of the cell cycle control gene TP53 are associated with early highly aggressive relapse with poor outcomes [48].

Our experiments revealed a distinct biphasic response in ALL cells upon BMSC-CM stimulation, with initial upregulation of AKT activity and intracellular ROS levels, followed by decreased pAKT and ROS level after 24 to 72 hours, together with increased antioxidants expression. Short-term drug resistance, characterised by increased AKT activity and intracellular ROS levels, was reversed by pretreating cells with PI-3K/AKT inhibitor, as previously reported [7, 8]. Similar to the long-term drug resistant phenotype, leukaemia-initiating cells have low AKT activity [49], as do ‘stem-cell like’ cancer drug resistant cells [25]. So the metabolic phenotype of drug resistant cells observed in this study is common with other drug resistant cancer cells and cancer stem cells [15, 50–52]. The association between short- and long-term drug resistances indicates an evolutionary process, as illustrated in our mechanistic model (Figure 8).

This process also leads to the upregulation of the mitochondrial anti-apoptotic protein MCL-1. MCL-1 is one of the most highly amplified genes in a broad range of human cancers [33, 53] and its expression rapidly changed in responds to cellular stresses [54]. A recent study suggested that outer mitochondrial membrane localized MCL-1 possess antiapoptotic activity, and the mitochondrial matrix localized MCL-1 isoform supports oxidative phosphorylation, ATP production, and the maintenance of mitochondrial membrane potential [55], so it is possible that inhibition of both the anti-apoptotic and the mitochondrial functions of MCL-1 may lead to more effective therapies. In line with these findings, we found that MCL-1 as the most upregulated anti-apoptotic molecules in cells exposed to CM. In some of our experiments, MCL-1 protein appeared as a doublets as detected by Western blot, depending on the conditions of SDS-PAGE. Suppression of MCL-1 expression by siRNA or obatoclax sensitized ALL cells to H_2_O_2_ induced cell death. PEITC, PL, or Adaphostin treatments effectively inhibited or abolished MCL-1 expression, but not that of BCL-2 or BCL-xL. Moreover, at certain concentrations, the multiple drug resistant SupB15^MR^ was more sensitive to PEITC or PL than its drug sensitive counterpart. Given that low MMP and high MCL-1 is the feature of BMSC mediated drug resistance, the extensive metabolic reprogramming in cancer cells may make them more vulnerable to motochondrial perturbations than normal cells [56], so it is possible that drug resistant cells may rely on high antioxidants and MCL-1 to maintain normal mitochondrial morphology. Inhibiting MCL-1 and antioxidants activity is able to further decrease MMP and eventually induce mitochondrial membrane collapse and cell death.

Our study has a number of limitations, chief of which is that the mechanisms of bone marrow microenvironment/BMSC mediated drug resistance were mainly explored in leukemic cell lines and murine models. Among the multiple signal pathways regulated by BMSC cells, as shown in our phosphoproteomics and gene expression analyses, we only investigated PI3K/AKT, cell proliferation, anti-apoptosis, and ROS metabolism in ALL cells. The nature of the subpopulations of cells that undergo redox adaptation remains unclear. As MRD levels vary with cytogenetic subtypes, it is possible that primary and secondary genetic subclonal changes may facilitate the redox adaptation. Murine models suggest that leukemic cells persist within the marrow after chemotherapy [57]. However the leukemic marrow environment is different to that of a marrow in remission. We do not know if the oxidative stress and response is different, or with time epigenetic transfer decreases leading to proliferation and increased chemosensitivity in daughter cells. Given the complexity of EMDR, more detailed investigation on other signalling pathways and better animal studies are required. Another limitation of this study is that the role of the immune system has not been explored *in vitro* and *in vivo* experiments. Genetic and epigenetic alterations happened in cancer cells may provide a diverse set of antigens that could be served as targets for immunotherapy. For example, PD1 pathway, an immune-checkpoint pathway that primarily operates in the tumour microenvironment, has emerged as a promising target in cancer [58].

Our study adds to the evidence that drug resistance in cancer cells is determined by both intrinsic and microenvironmental factors. Further in-depth investigation of metabolic profiling and epigenetic changes in the development of cancer drug resistance will provide insights into clinical treatment failure and relapse.

## MATERIALS AND METHODS

### Human tissue and cell lines

Clinical samples were obtained after informed signed consent from patients enrolled into the national clinical trial (ALL 2003) for children with ALL. National and Local ethical committees approved analyses of samples and ASNase activity. Primary ALL blast cells from bone marrow aspiration were separated with Ficoll gradient centrifugation (Lymphoprep; Axis-Shield). Leukemia cell lines were maintained in RPMI 1640 (Lonza, Belgium) supplemented with 10% fetal calf serum (FCS, Biosera). Primary BMSCs were prepared from ALL patients at diagnosis or have been received intensive chemotherapy. Only BMSC of three to six passages were used. Primary BMSC and human BMSC cell line HS-5 and hTRET-MSC were cultured in DMEM medium (Lonza) supplemented with 10% FCS, 100 U/ml penicillin and 100 μg/ml streptomycin. Differentiation of BMSC into osteoblast-like cells was assessed by alkaline phosphatase (ALP) staining (Roche, Germany) and osteopontin expression.

### Animal experiments

All animal procedures were approved by the Cancer Research UK Manchester Institute's Animal Ethics Committee and performed under a project license issued by the United Kingdom Home Office, in keeping with the Home Office Animal Scientific Procedures Act, 1986. NOD/SCID/IL-2Rγ^null^ (NSG) mice were purchased from Jackson Laboratories (Bar Harbor, ME, USA) and bred in house. To investigate bone marrow microenvironment mediated redox adaptation in ALL cells, 1 × 10^5^ of ALL cells were transplanted into NOD/shi-scid/IL-2Rγ^null^ mice femoral bone marrow. Mice were treated with methotrexate (5 mg/kg body weight) or PBS after 8 days transplantation. Bone marrow mononuclear cells were flushed from bone marrow after three weeks' transplantation. Cells were gated with GFP (for SupB15^LucGFP^ cells) or CD45 (for non-labelled cells) on flow cytometry.

### Generation of BMSC derived extracellular matrix (ECM)

BMSC-ECM was generated by growing BMSC to confluence in normal medium [24]. Briefly, primary or cell line BMSCs were cultured in normal medium, just prior to reaching confluent, cells were treated with 50 ug/ml of ascorbic acid (Sigma) and cultured for another 5 days. BMSCs layer were treated with extraction buffer containing 0.5% (v/v) Triton X-100 and 20 mM NH4OH, gave rise to cell free ECM that remain attached to the culture plates. After washed three times with ice-cold PBS, the resulting extracted ECM can be stored at 4°C in PBS containing 100 U/ml penicillin and 100 ug/ml streptomycin with parafilm sealing for up to 3 months.

### Generation of conditioned medium and ultrafiltration

BMSCs were cultured in complete medium on the BMSC-ECM layer until reach about 70% confluence, then serum free RPMI1640 were added and incubated for 24–48 hours. Cell culture supernatant were collected, centrifuged at 3500 rpm for 10 min to clear cells debris, then passed 0.2 μm filter. CM was snap frozen in dry ice, and kept in −80°C. CM was diluted with normal medium at a ratio of 1:1 just before use. CM fractions (<3kDa and >3kDa) were ultra-filtrated at 3000 Da (Millipore, Billerica, MA, USA), 3000 × g, 60 min, at 4°C.

### RNA extraction, microarray, and GSEA analysis

Total RNA were purified by Qiagen RNeasy Micro kit, cDNA was amplified and labelled with Nugen Ovation Biotin labelling system (NuGEN Technologies). Labelled probes were hybridized to Affymetrix U133A 2.0 PLUS Array. The probe-level expression values in the data obtained from the Affymetrix HG133plus2 array were normalised and summarised as the expression values at the probeset level using the RMA method implemented in the Affymetrix's tool ‘apt-probeset-summarize’. Data (.CEL files) were analyzed using the R statistical package bioconductor and Genespring 7.3.1 software. Gene array data were deposited in NCBI gene expression database Gene Expression Ominibus (GEO), accession number GSE48876.

The probe set-level expression data was loaded into the gene set enrichment analysis (GSEA) software. We created six gene sets, which involved in ROS metabolism (68 genes), AKT pathway (64 genes), apoptosis (110 genes), ATPase (46 genes), DNA repair (41 genes) and Chromatin remodelling (76 genes) by selecting genes from previously published gene list or the gene lists from SABiosciences website (http://www.sabiosciences.com/ArrayList.php) with modification based on published methods, only genes with published evidence of functions were included in the final gene list. In the GSEA analysis, we used the default settings except using the ‘gene-set’ as the permutation type, the Chip file provided by the GSEA software for the Affymetrix HG133plus2 array, and the ‘Diff_of_Classes’ option as the metric for ranking genes.

### Triple SILAC based phosphoproteomics

SupB15 and HS-5 cells were grown in heavy (H) Arginine (^13^C_6_^15^N_4_ L-Arginine-HCl)/Lysine (^13^C_6_^15^N_2_ L-Lysine-2HCl), intermediate (I) Arginine (^13^C_6_ L-Arginine-HCl)/Lysine (^13^C_6_ L-Lysine-2HCl) and light (L) Arginine (L-Arginine-HCl)/Lysine (L-Lysine-2HCl) media for a minimum of five passages according to manufacturer's instructions. All media was supplemented with 230 μg/ml L-Proline to prevent conversion of Arginine to Proline. After serum starved overnight, SupB15 cells were treated with SILAC conditioned media (CM) from HS-5 cells for 30 mins or 2 hrs. Then cells were washed with ice-cold PBS, equal numbers of H, I and L viable SupB15 cells were mixed together, lysed with 50 mM Tris, 4% SDS containing protease and phosphoprotease inhibitor cocktails (Roche) on ice for 30 mins. Protein samples were then digested with trypsin, desalted and dried down. Seven aliquots (each around 320 ug) were combined. The sample was split equally into ten portions for TiO2 enrichment and subjected to a LTQ OrbitrapXL mass spectrometer (Thermo Fisher, Hertfordshire, UK) for analysis.

### shRNA and siRNA transfection

Lentiviruses which express GFP, p27 shRNA or their empty vector control were produced in 293T cells, transfected into SupB15 cells and stable transfection were selected with 10 ug/ml of Puromycin. Accell MCL-1 siRNA or its control siRNA (Thermo Scientific Dharmacon) were transduced into ALL cell lines according to manufacturer's instruction. 3 days after transfection, protein knockdown were assessed by Western Blot and cells were immediately used for experiments.

### Cell proliferation and viability

Cell proliferation and viability was analyzed by using CellTiter96Aqueous One Solution Cell proliferation Assay kit (Promega, Madision, WI, USA) according to the manufacturer's protocol. Cell viability was determined by recording the absorbance at 490 nM using a 96-well plate reader (FLUOstar Omega). Cell viability was also be determined by Trypan blue exclusion (Invitrogen) assay and the cell viability were assessed in a TC10 Automated cell counter (Bio Rad, Berkeley, CA, USA).

### Immunoblots and antibodies

Cells were suspended in lysis buffer (20 mM Tris-HCl, pH 7.8, 50 mM NaCl, 5 mM EGTA, and 1% v/v Triton X-100) containing freshly added protease and phosphatase inhibitors (Roche). Lysates were clarified by centrifugation at 4°C, and protein concentration was determined by Bio-Rad protein assay.

To verify the protein contents of the BMSC-ECM after alkaline detergent extraction, BMSC-ECM were scratched from culture plate and dissolved in buffer containing 100 mM Tris-HCl (pH 6.8), 200 mM dithiothreitol and 4% SDS. Samples were homogenized by pass through 27 g needle for at least 7 times, incubated on ice for 60 minutes, cleared by centrifugation and subjected to protein concentration assessment. Then 0.2% glycerol and 0.2% bromophenol blue (final concentration) were added and samples were heated at 95°C for 5 minutes.

The following antibodies are used: phosphor-AKT Ser 473 and were from Cell Signalling. γ-H2AX, osteopontin, β-actin, MnSOD2, BCL-2, and BCL-xL were from Millipore. p27, p21, collagen 1, fibronectin, MCL-1, Gpx1/2, were from Santa Cruz Biotechnology.

### Flow cytometry

ROS level (CM-H2DCFDA or CellROX™ Deep Red), Δψm (Mitotracker CMXRos), and Mitochondrial Ca^2+^ concentration (Rhod2-AM) were analyzed using a FACSCalibur flow cytometer (BD Bioscience, Oxford, UK). Intracellular GSH level were assessed in a LSR model II flow cytometry (BD Bioscience, Oxford, UK) with ThiolTracker™ Violet dye staining. The fluorescence dyes were from Invitrogen (Carlsbad, CA, USA), and data were analyzed using FlowJo software (FlowJo, Oregon, USA). Cell isolation by flow sorting was performed on a BD FACS Aria III (BD Bioscience).

### Measurement of calcium concentration

The concentration of Calcium ions was measured by using Calcium Colorimetric Assay Kit (BioVision, Milpitas, CA, USA) and determined by recording the absorbance at 575 nm using a 96-well plate reader (FLUOstar Omega, BMG LABTECH, Germany).

### Statistical analysis

Statistical analyses were performed using unpaired 2-tailed Student's *t* test for *in vitro* cell line data; non-parametric Mann-Whitney test for *in vivo* and patients sample data. Statistical calculations were performed with GraphPad Prism software. *P* value < 0.05 was considered as significant. Data are expressed as means ± SEM. *p < 0.05, **p < 0.01, ***p < 0.001.

## SUPPLEMENTARY FIGURES AND TABLES








