# Supplementary material for: Stromal cell-mediated mitochondrial redox adaptation regulates drug resistance in childhood acute lymphoblastic leukemia
Source: Oncotarget. 2015 Oct 13;6(40):43048–64. doi: 10.18632/oncotarget.5528 (PMC4767490; doi:10.18632/oncotarget.5528)
Supplement: Supplementary file 1 [file oncotarget-06-43048-s001.pdf]

SUPPLEMENTARY METHODS

Microarray data analysis

The gene expression data sets were analysed using GeneSpring GX software (Agilent Technologies, Inc.). Gene expression data were summarized by multichip averaging algorithm. Probe sets with signal intensities in the lowest 20 percentile of all intensity values were removed. The gene expression data were incorporated with Benjamini–Hochberg FDR multiple testing corrections, then subjected to ANOVA analysis. A p-value ≤ 0.05 were regarded as differentially expressed genes. Probe sets were further filtered on the basis of a fold-change cut-off of ≥ 2.0.

Functional analysis

Ingenuity Pathway Analysis (IPA) software was used to conduct functional annotation of the differentially expressed genes. Genes that met the p-value cut-off of ≤ 0.05 and fold-change cut-off of ≥ 2.0 were associated with biological functions and/or diseases in Ingenuity's knowledge base. Right-tailed Fisher's exact test was used to determine the association between each biological function and/or disease and gene expression data set.

Canonical pathway analysis

The significance of the association between the gene expression data set and the canonical pathway was measured in two ways: (1) a ratio of the number of molecules from the data set that map to the pathway divided by the total number of molecules that map to the canonical pathway, and (2) the association between the genes in the data set and the canonical pathway was determined by Fisher's exact test, p-value ≤ 0.05 as significant.

Hierarchical clustering was performed on specific gene sets selected from the functional annotation data by complete linkage and uncentered correlation using software Cluster 3.0. Results were visualized using Java TreeView.

SUPPLEMENTARY FIGURES AND TABLES

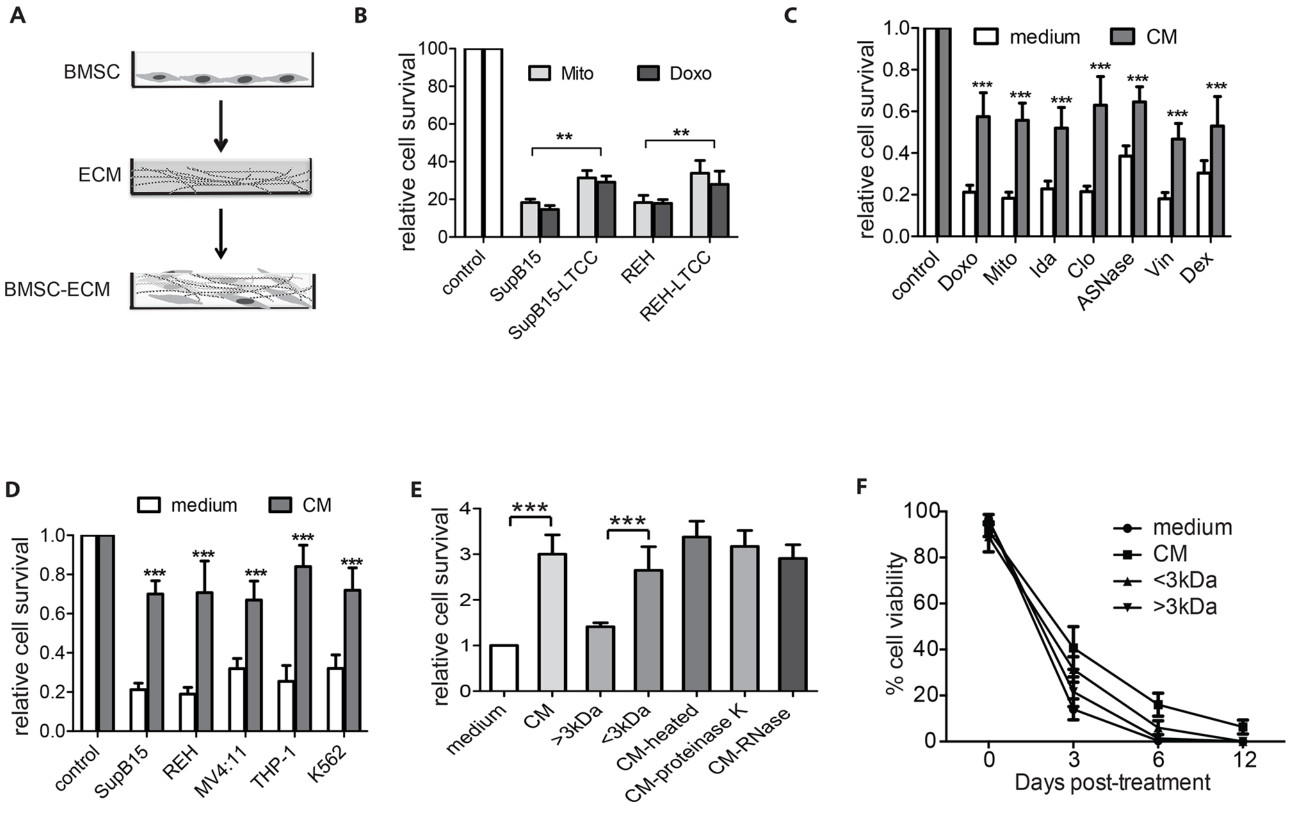

Supplementary Figure S1: Generation of multidrug resistant subpopulations from ALL cell lines in a BMSC-ECM culture model. A. Schematic showing generation of BMSC derived ECM scaffold and the BMSC-ECM culture system. **B.** LTCC cells (SupB15 or REH cells co-cultured with HS-5 at a ratio of 20:1 for 3 months) are more resistant to doxorubicin (Doxo, 50 nM) or mitoxantrone (Mito, 10 nM) after 3 days' treatment. **C.** SupB15 cells were incubated in normal medium or CM, treated with doxorubicin (Doxo, 50 nM), mitoxantrone (Mito, 10 nM), idarubicin (Ida, 100 nM), clofarabine (Clo, 300 nM), vincristine (Vin, 10 nM), and dexmethasone (Dex) for 3 days, or asparaginase (ASNase, 0.5 U/ml) for 4 days, untreated cells as control. **D.** Leukemic cells SupB15, REH, MV4:11, THP-1 and K562 cells were in incubated in normal medium or CM and treated with Doxo (50 nM) for 3 days. **E.** SupB15 cells were cultured in normal medium; CM from HS5; >3kDa fraction of HS5-CM; <3kDa fraction of HS5-CM; CM has been heated at 95°C for 10 minutes (CM-heated); CM has been treated with 50ug/ml Proteinase K at 50°C for 1 hour, then heated at 95°C to inactive Proteinase K (CM-proteinase K); CM has been treated with RNase A (5 IU/ml) for 1 hour at 37°C, then treated with 50 ug/ml Proteinase K at 50°C for 1 hour to inactive RNase, followed by heated at 95°C for 10 minutes to inactive Proteinase K (CM-RNase). Cells were treated with Mito (10 nM) for 3 days. Cell viability was determined by MTS assay, and data were presented as fold change comparing cells cultured in normal medium. **F.** SupB15 cells were cultured in normal medium; CM from HS5; >3kDa fraction of HS5-CM; and <3kDa fraction of HS5-CM, then treated with Mito (10 nM) for 6 days. Cells were then transferred into drug-free medium for another 6 days. Cell viability was assessed with Trypan blue exclusion assay.

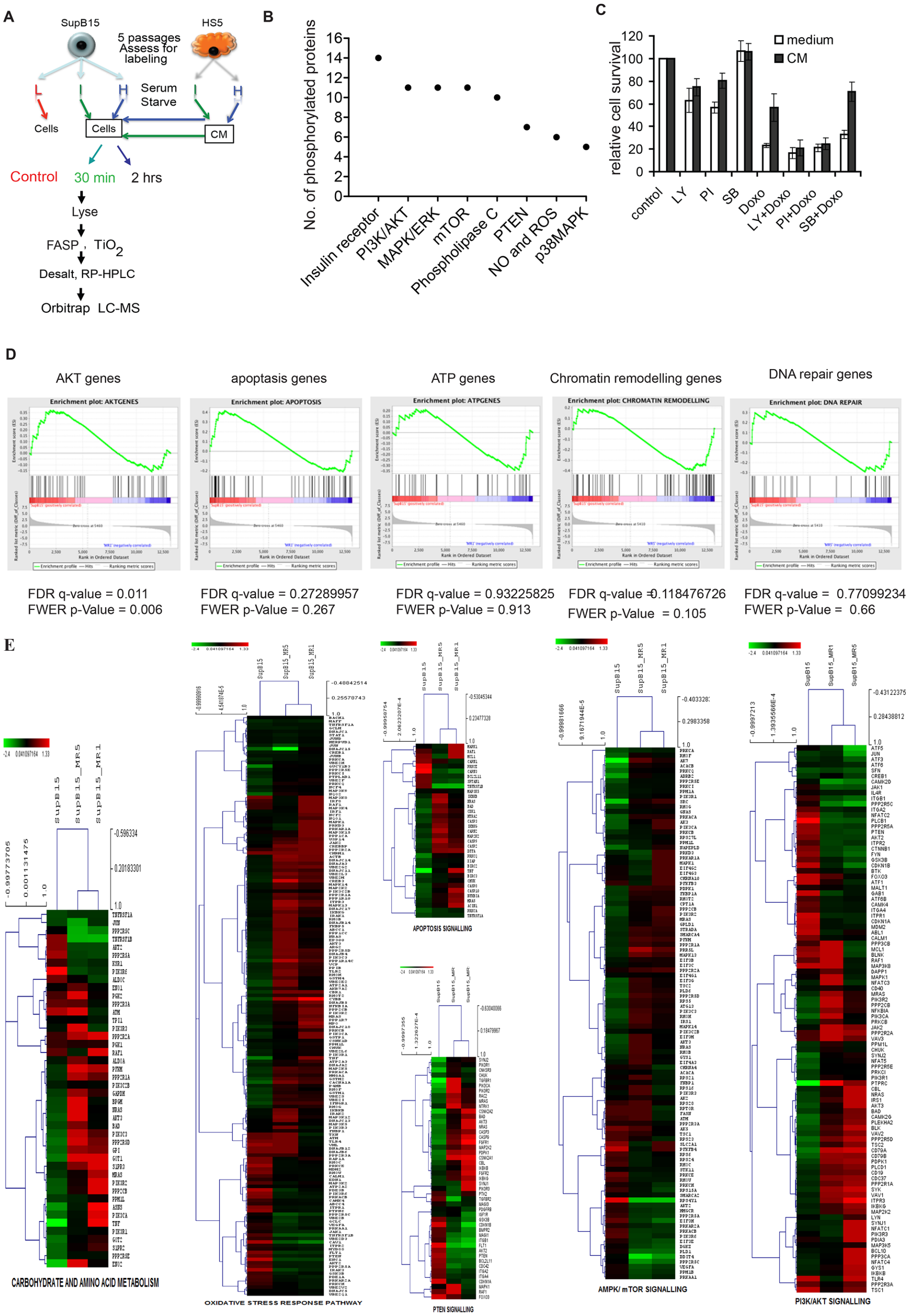

Supplementary Figure S2: Involvement of PI3K/AKT and ROS pathway in BMSC mediated drug resistance in ALL. A. Schematic showing SILAC labeling and downstream analysis. SupB15 cells exposed to HS5-derived CM for 0.5 or 2 hours. H, as heavy: Arginine ($^{13}C_6$ $^{15}N_4$ L-Arginine-HCl)/Lysine ($^{13}C_6$ $^{15}N_2$ L-Lysine-2HCl); I, as intermediate: Arginine ($^{13}C_6$ L-Arginine-HCl)/Lysine ($^{13}C_6$ L-Lysine-2HCl); and L, as light: Arginine (L-Arginine-HCl)/Lysine (L-Lysine-2HCl). FASP, Filter-Aided Sample Preparation. Tio2, titanium dioxide. LC-MS, liquid chromatography–triple quadrupole mass spectrometry **B.** Results of phosphoproteomics analysis showing the signaling pathways activated in SupB15 cells after incubated in CM for 2 hours. **C.** SupB15 cells were incubated in medium, treated with PI3K/AKT inhibitor LY294002 (LY, 20 µM) and PI103 (PI, 10 µM), p38MAPK inhibit SB203580 (SB, 25 µM) and Doxo (40 nM) for 3 days; or pretreated with LY, PI, or SB for 2 hours, then added Doxo (40 nM) for 3 days. Cell survival was assessed with MTS assay. Non-treated cells as control. **D.** GSEA plots showing differential expression of AKT, apoptosis, chromatin remodeling, DNA repair, and ATPase related genes in SupB15 and SupB15^{MR} cells. **E.** Heat maps showing the differential gene expression related to oxidative stress, apoptosis, PTEN, PI3K/AKT, AMPK/mTOR and carbohydrate and aminoacid metabolism pathways. (*Continued*)

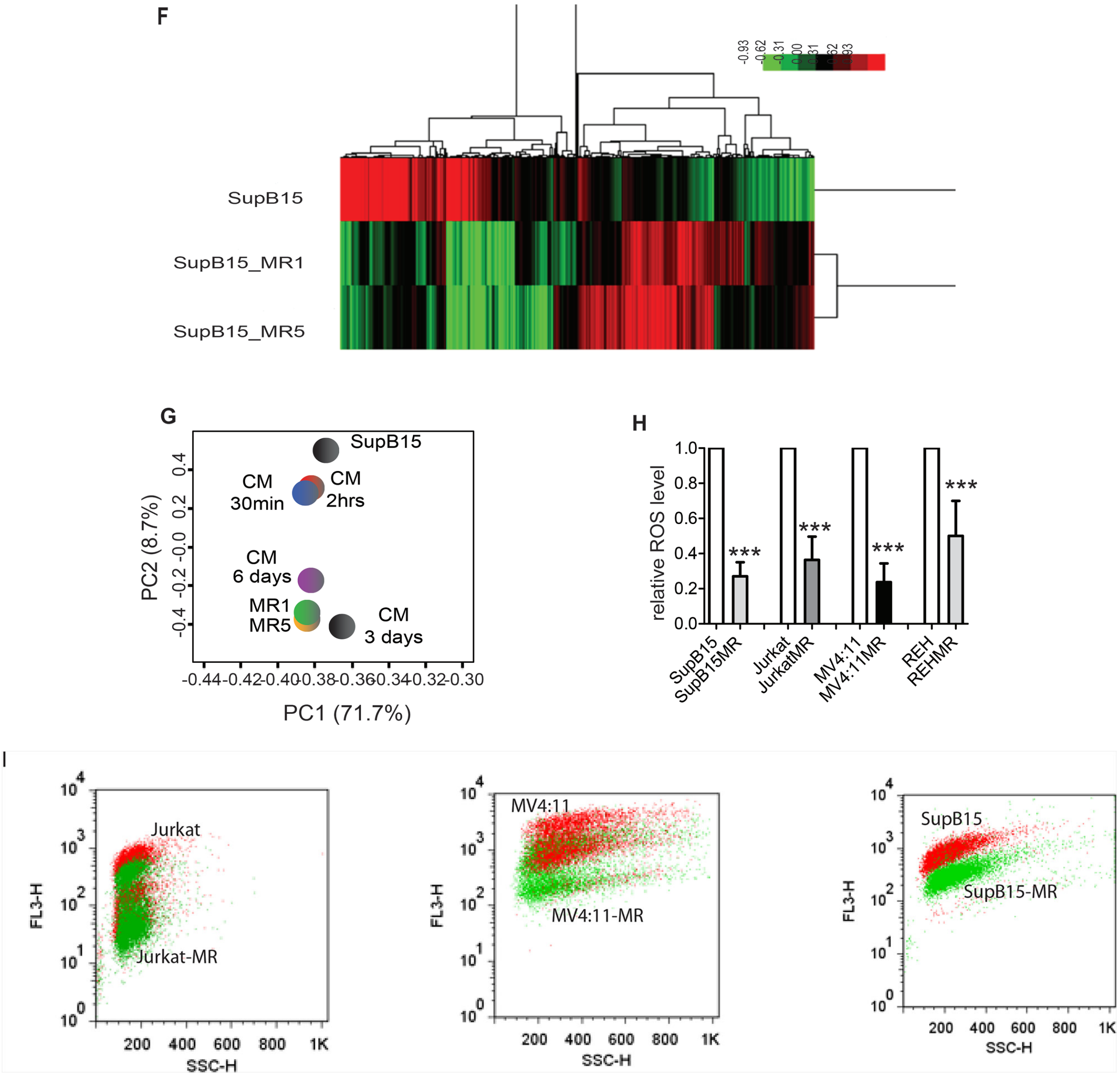

Supplementary Figure S2: (*Continued*) Involvement of PI3K/AKT and ROS pathway in BMSC mediated drug resistance in ALL. F. Heat map showing the differential gene expression between SupB15 and two MR clones SupB15-MR1 and MR5. **G.** PCA analysis of the protein-coding genes from Affymetrix U133A 2.0 PLUS Array obtained from SupB15, SupB15 exposed to CM for varying times and SupB15 clones (MR1 and 5), computed by using the R function princomp on the expression (log_2 scale). **H.** SupB15, Jurkat, MV4:11, and REH cells, or their drug resistant subclone MR cells were incubated in normal medium. Intracellular ROS level were determined by DCF-DA staining and followed with flow cyetometry. **I.** Histograms show that the MR cells have lower mitochondrial membrane potential (CMXRos) than their drug sensitive parent cells.

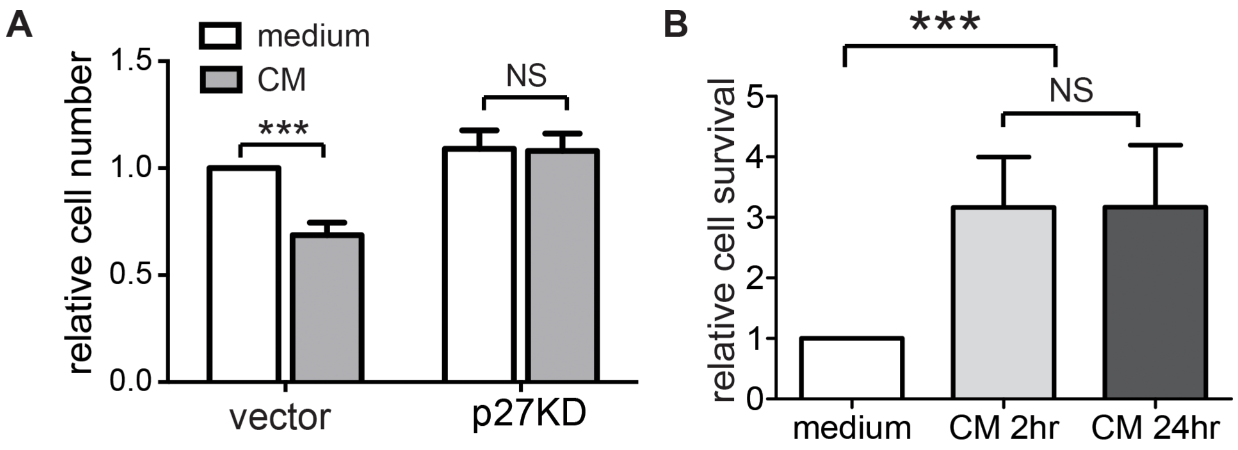

Supplementary Figure S3: A. SupB15 cells with p27 wild type (vector) or knockdown (p27KD) were incubated in normal medium or CM for 3 days. CM inhibited SupB15^{vector} cell proliferation, but not SupB15^{p27KD} cells. **B.** SupB15 cells were cultured in (1) medium; (2) CM for 2 hours; and (3) CM for 24 hours, then treated with Mito (10 nM) for 3 days. Cell survival was assessed by MTS assay.

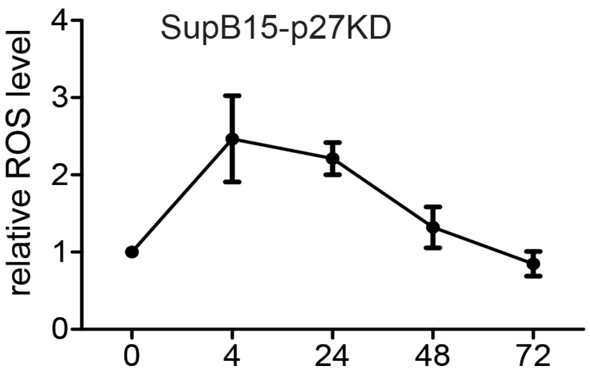

Supplementary Figure S4: Levels of ROS (DCF-DA) in SupB15 with p27 knockdown cells after incubation in CM for up to 72 hours.

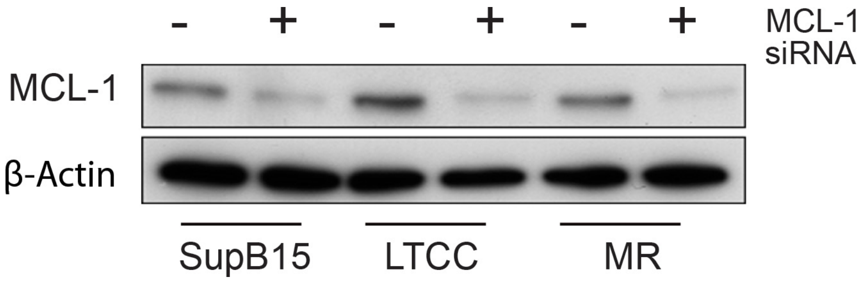

Supplementary Figure S5: Immunoblot validation of MCL-1knockdown by siRNA transfection in SupB15, SupB15^{LTCC} and SupB15^{MR} cells.

A

Number	Sex	Immuno	Age (Yrs)	WBC $(x10^9/L)$	MRD (d28)	Asparaginase Activity TP1 (d16)	TP2 (d28)
5919	F	B	4	4.28	Low Risk	adequate	adequate
6108	M	B	3	23.6	Low Risk	adequate	adequate
6489	M	B	2	36.8	Low Risk	adequate	adequate
6339	F	B	2	146	Low Risk	adequate	adequate
6841	F	B	7	35	Low Risk	adequate	adequate
6941	M	B	3	10.4	Low Risk	adequate	adequate
6551	M	B	2	103.8	Low Risk	adequate	adequate
5641	M	B	4	17.9	High Risk	adequate	adequate
6707	M	B	6	6.6	High Risk	adequate	adequate
6907	M	B	11	6.4	High Risk	adequate	adequate
6167	F	B	4	15.2	Low Risk	adequate	adequate
6241	M	B	5	17.8	Low Risk	adequate	adequate
6571	M	B	2	60.1	Low Risk	adequate	adequate
6163	M	B	10	3.2	Low Risk	adequate	adequate
6241	M	B	5	17.8	Low Risk	adequate	adequate
6167	F	B	4	15.2	Low Risk	adequate	adequate
6162	F	B	2	41.02	High Risk	adequate	adequate
6566	F	B	5	17.9	High Risk	adequate	adequate
6531	M	B	3	186.2	High Risk	adequate	adequate
6326	M	B	1	75	High Risk	adequate	adequate
6777	F	B	7	3.9	Low Risk	adequate	adequate
6989	F	B	9	8.5	High Risk	adequate	adequate
6330	M	N	11	580	High Risk	adequate	adequate
6481	F	B	3	13.7	Low Risk	adequate	adequate
6769	F	B	4	32.1	High Risk	adequate	adequate
6355	F	B	2	103.8	Low Risk	adequate	adequate

B

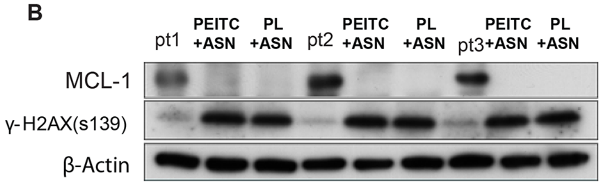

C

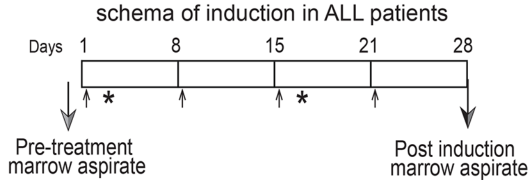

Supplementary Figure S6: A. Clinical demographics of the patients used in the study, total 27 patients. **B.** Schematic showing first 4 weeks of therapy and sample collection in children with ALL treated on the UKALL 2003 protocol. Arrow: Vincristine; Asterisk: PEG-ASNase. **C.** Expression of MCL-1 and γ-H2AX in primary ALL blasts from 3 patients (pt1, pt2 and pt3) after treatment: PEITC (2 μM) for 48 hours; ASNase (ASN) for 72 hours (2 IU/ml for REH, 5 IU/ml for SupB15[MR]); or ASNase for 24 hours, then PEITC for further 48 hours. β-Actin as loading control.

Supplementary Table S1: phosphoproteomics data.

Supplementary Table S2: Gene lists for GSEA analysis.

Supplementary Table S3: Ingenuity Canonical pathways.
